# Nomogram Models for Predicting Risk and Prognosis of Newly Diagnosed Ovarian Cancer Patients with Liver Metastases - A Large Population-Based Real-World Study

**DOI:** 10.7150/jca.64255

**Published:** 2021-10-25

**Authors:** Gui-Min Hou, Chuang Jiang, Jin-peng Du, Chang Liu, Xiang-zheng Chen, Ke-fei Yuan, Hong Wu, Yong Zeng

**Affiliations:** 1 Department of Liver Surgery & Liver Transplantation, State Key Laboratory of Biotherapy and Cancer Center, West China Hospital, Sichuan University, Chengdu, China.; 2 Laboratory of Liver Surgery, West China Hospital, Sichuan University, Chengdu, China.; 3 Collaborative Innovation Center of Biotherapy, Chengdu, Sichuan Province, China.

**Keywords:** Ovarian Cancer, Liver Metastases, prevalence, nomogram, SEER

## Abstract

**Background:** Previous studies about liver metastases (LM) in newly diagnosed ovarian cancer (NDOC) patients based on Surveillance, Epidemiology, and End Results (SEER) program disregarded selection bias of missing data.

**Methods:** We identified Data of NDOC patients from SEER between 2010 and 2016, presented a comprehensive description of this dataset, and limited possible biases due to missing data by applying multiple imputation (MI). We determined predictive factors for underlying LM development in NDOC patients and evaluated prognostic factors in NDOC patients with LM (OCLM). We then established predictive nomograms, assessed by the concordance index, calibration curve, decision curve analysis (DCA), and clinical impact curves (CIC).

**Results:** The amount of missing data for different variables in SEER dataset ranges from 0 to 36.11%. The results between complete dataset and MI datasets are similar. LM prevalence in NDOC patients was 7.18%, and median overall survival for OCLM patients was 11 months. The C-index of risk nomogram for LM development in the training cohort (TC) and validation cohort (VC) were 0.764 and 0.759, respectively. The C-index and integrated area under curve within five years of prognostic nomogram for OCLM patients in the TC and VC were 0.743 and 0.773, 0.714 and 0.733, respectively. For both nomograms, DCA revealed favorable clinical use and calibration curves suggested good consistency.

**Conclusion:** The risk nomogram is expected to aid clinicians in identifying high-risk groups of LM development in NDOC patients for intensive screening. The prognostic nomogram could facilitate individualized prediction and stratification for clinical trials in OCLM patients.

## Introduction

Ovarian cancer (OC) is the most lethal form of malignancy in the female genital system in the United States, with an estimated 22,530 newer cases and 13,980 deaths annually 1. Approximately 75% of all OC patients remain undiagnosed until the advanced stage, while 12-33% of these cases are diagnosed with distant metastases 2,3. The liver is the most common site for distant metastasis in OC, followed by distant lymph nodes, lung, bone, and brain 4-6.

Population-based studies had reported the prevalence of liver metastases (LM) in newly diagnosed OC (NDOC) patients; however, results are inconsistent 5,7. Although previous studies identified the hazards associated with underlying LM development in NDOC patients, the tool quantifying risk of LM development is unavailable. Albeit prior studies determined prognostic factors for NDOC patients with LM (OCLM), a predictive model needs to be further investigated to facilitate clinical use 5,7. Furthermore, missing data problems in population-based databases had been disregarded in previous studies 5,7, which might introduce bias and even incorrect conclusions.

Therefore, we described missing data and possible bias based on the Surveillance, Epidemiology, and End Results (SEER) program of the National Cancer Institute. Then, we provided a dataset that limited selection bias. Finally, we evaluated epidemiology and risk factors associated with LM development in NDOC patients, revealed prognostic predictors for OCLM patients. Furthermore, we established and validated nomogram models to aid clinical practice during diagnosis and treatment of OCLM patients.

## Methods

### Data Sources and Patients

We queried the SEER database from 2010 to 2016 since no metastatic information was recorded before 2010. The site code of ICD-O-3 (International Classification of Diseases for Oncology-3)/WHO 2008 was restricted as “Ovary”. Patients with histologically confirmed ovarian cancer were included and the exclusion criteria were as follows: patients aged below 18 years, diagnosed with carcinoma *in situ*, benign or borderline tumors, diagnosed at autopsy or via death certificate, those with no information on LM or follow-up. A detailed summary of selection criteria is illustrated in Supporting Figure 1. Finally, we identified a total of 37,500 eligible NDOC patients, of which 2,691 cases were diagnosed with LM. All 37,500 NDOC patients were used in establishing and validating the risk nomogram for LM development. Prognostic nomogram was established and validated in the 2,691 OCLM patients.

### Covariates

Variables such as age, race (White, Hispanic, Black, and Asian), marital status (married or unmarried), insurance status (uninsured or insured), tumor laterality (left, right, and bilateral), T stage (T1, T2, or T3), N stage (N0 or N1), tumor grade (I/II, III, or IV), histology (serous or non-serous), cancer antigen 125, (CA 125, normal and elevated), lung, bone, or brain (LBB) metastases, and primary cytoreduction surgery (CRS) (optimal: residual focus < 1 cm, suboptimal: residual focus > 1 cm, or no CRS) were used for constructing the risk and prognostic models. OS was defined as the time between initial diagnosis to death or last follow-up (December 31, 2016).

### Statistical analysis

The pattern of missing data was visually displayed based on VIM and mice package in R. To maximize statistical power and minimize bias that results from exclusion of missing data, multiple imputation (MI) based on five replications and a chained equation approach method was used to account for missing data of variables in SEER 8, including marital status, CA 125 levels, tumor laterality, race, grade, T stage, N stage, and LBB metastases, insurance, and CRS.

Categorical variables were grouped based on clinical considerations and previous literature 5,9, which were decided before modeling. Continuous variables were presented as means ± standard errors (SD) or medians with interquartile ranges (IQR), differences between groups were analyzed using a *t* test. Categorical variables were presented as numbers and percentages and analyzed using either Pearson's X^2^ or Fisher's exact tests. Survival analyses were performed using the Kaplan-Meier method and tested using the Log-rank test.

In a complete cases dataset, which excluded cases with missing data of variables, all NDOC patients as well as OCLM patients, were randomly divided into the TC and VC (2:1). NDOC patients and OCLM patients in the TC were subjected to univariate regression analysis, variables with P < 0.1 in univariate analysis and clinically relevant were incorporated into multivariate analyses. Both the risk and prognostic nomograms were formulated based on results from the final multivariate regression models by backward stepwise selection with the Akaike Information Criterion (AIC) 10. As a sensitivity analysis, the procedure was repeated in the 5 MI datasets, and the regression coefficients and standard errors were pooled to evaluate bias secondary to missing data 11.

We applied C-index, calibration curves and Hosmer-Lemeshow goodness of fit test, decision curve analysis (DCA) and clinical impact curves (CIC) to evaluate the risk nomogram for LM development in NDOC patients. Meanwhile, we used C-index, calibration curves, the AUC of time-dependent receiver operating characteristics (td-ROC) 12 and DCA curves to appraise the prognostic nomogram for OCLM patients. The integrated AUC was defined as the mean of the AUC values of prognostic nomogram within five years. All analyses were conducted using the SPSS version 17 (IBM, Armonk, NY, USA) and R version 4.0.2 (http://www.r-project.org). Statistical tests were 2-sided, with data followed by P < 0.05 considered statistically significant.

## Results

### Sociodemographic characteristics and prevalence of LM in NDOC patients

Screening the SEER database revealed a total of 37,500 eligible NDOC patients. Among them, 2,691 cases, accounting for approximately 7.18% of the entire cohort, were diagnosed with LM (Supplementary Figure 1). A summary of sociodemographic and clinical characteristics of patients with or without LM is outlined in Table 1.

### Characteristics of missing data

The amount of missing data for different variables in SEER ranges from 0 to 36.11% in NDOC patients and ranges from 0 to 56.93% in OCLM patients, which were quantified and displayed in Supplementary Figure 2. We did not find an unusual pattern in missing data by visual inspection and categorized it as missing at random (MAR), which fits a necessary assumption for multiple imputation 13. Moreover, there was no statistical difference in baseline characteristics between observed complete cases data and imputation datasets (Table s1, Table s2), so further analyses were performed based on the complete dataset.

### Predictive factors for development of LM

**Figure 1:**
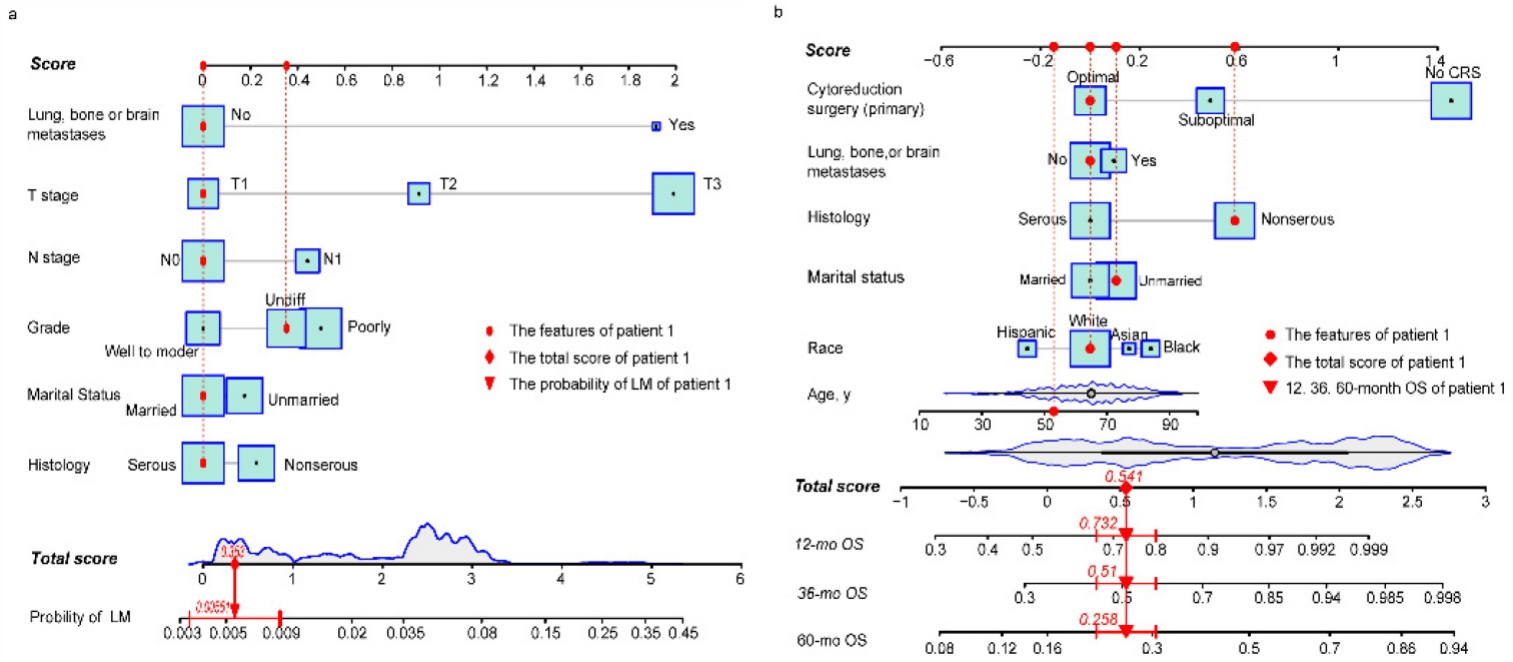
** Risk nomogram for LM development in NDOC patients and prognostic nomogram for OCLM patients. (a):** Risk nomogram for predicting LM development in NDOC patients. **(b):** Nomogram for predicting prognosis of OCLM patients. Points are assigned for all risk factors, first by drawing a line upward from the corresponding value to the "Score" line to get the points for each factor, then the points for all factors are added to obtain the total score and a vertical line is drawn to the “Total score” row to determine LM occurrence as well as 1-, 3- and 5-year survival rates. Patient 1 from this study is shown as an example (presented in red). The distinct area of rectangles represents the difference of the relative proportion of patients in each subgroup. The distribution of age, total scores of risk and prognostic nomograms are also shown in Figure 1a and 1b. LM: liver metastases. NDOC patients: newly diagnosed ovarian cancer patients. OCLM patients: NDOC patients with LM. 12 mo OS: 12-month overall survival.

Baseline characteristics of NDOC patients in the TC and VC groups were comparable (Table s3). Results from univariate logistic analysis in the TC (*n* = 24,718) revealed that age, marital status, CA 125 levels, tumor laterality, race, grade, T stage, N stage, and lung, bone, or brain (LBB) metastases were predictors for LM development. The nomogram model, after backward stepwise selection based on AIC, revealed the following as risk factors for LM incidence; unmarried (vs. married; OR, 1.19; 95%CI: 0.98-1.45; *p* = 0.084), non-serous (vs. serous; OR, 1.28; 95%CI: 1.02-1.61; *p* = 0.053), grade III (vs. grade I/II; OR, 1.61; 95%CI: 1.15-2.26; *p* = 0.004), grade IV (vs. grade I/II; OR, 1.42; 95%CI: 1.01-2.01; *p* = 0.048), T2 stage (vs. T1 stage; OR, 2.60; 95%CI: 1.48-4.56; *p* = 0.002), T3 stage (vs. T1 stage; OR, 7.50; 95%CI: 4.66-12.06; *p* < 0.001), N1 stage (vs. N0 stage; OR, 1.58; 95%CI: 1.29-1.93; *p* < 0.001), and LBB metastases (vs. no metastases to other distant organs; OR, 6.69; 95%CI: 5.12-8.73; *p* < 0.001) (Table 2). The most significant contributors were T stage and LBB metastases, followed by grade, N stage, histology and marital status (Fig. 1a). The pooled OR derived from 5 MI datasets gave similar results to OR of complete dataset (Table 2).

### Nomogram model predicts risk factors of LM development in NDOC patients

**Figure 2:**
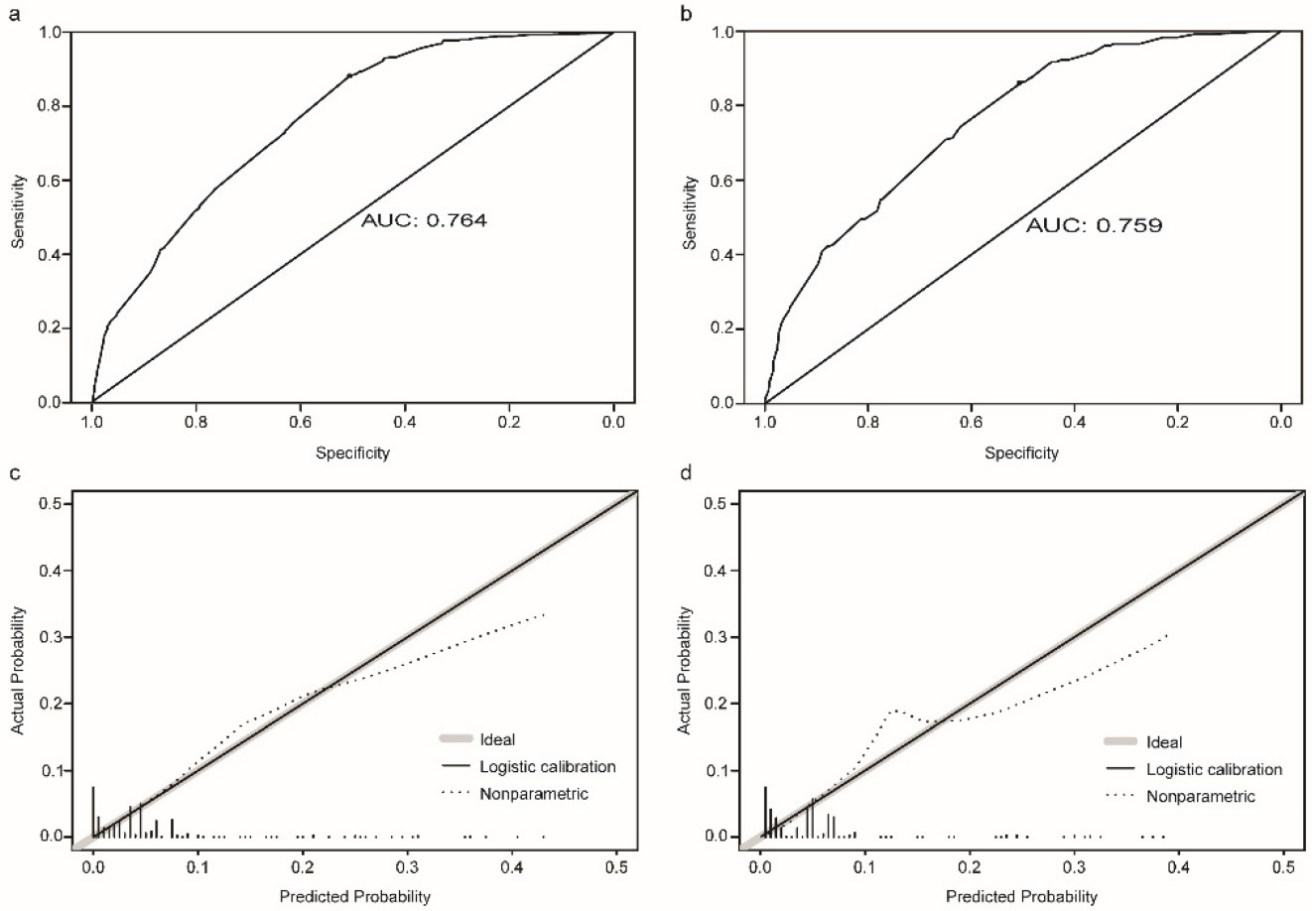
** Evaluation of the risk nomogram for LM development in NDOC patients. (a, b):** Receiving operating characteristic (ROC) curves in the TC (a) and VC (b); **(c, d):** Calibration plots in the TC (c) and VC (d). LM: liver metastases. NDOC patients: newly diagnosed ovarian cancer patients. TC: training cohort. VC: validation cohort.

**Figure 3:**
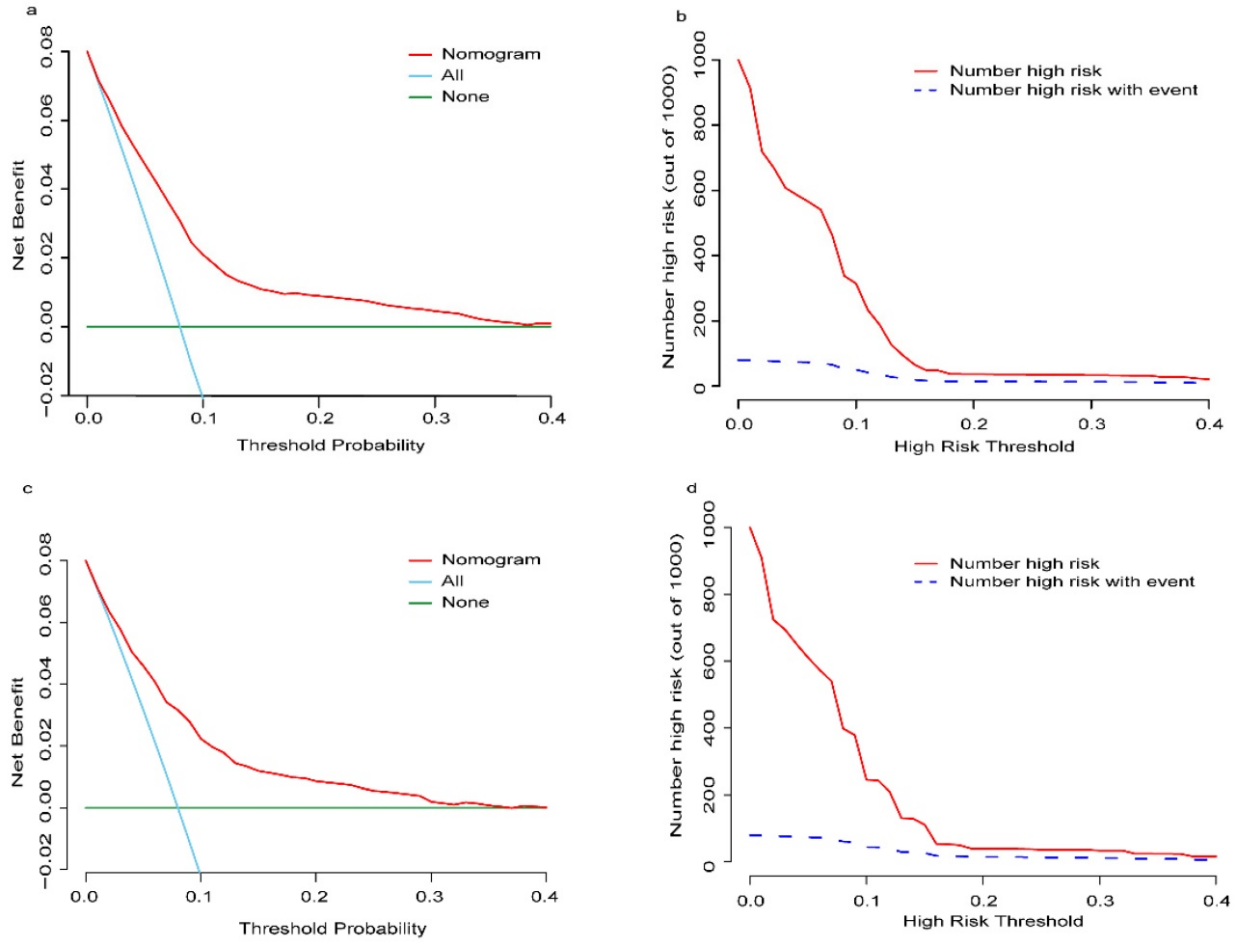
** Decision curve analysis (DCA) and clinical impact curve (CIC) of the risk nomogram for LM development in NDOC patients.** DCA and CIC of risk nomogram were calculated for both the TC and VC. In DCA, the net benefits (y axis) of risk nomogram were calculated. Horizontal red lines assume no cases will experience the event; green lines assume all cases will experience the event; blue lines represent the net benefits across a range of threshold probabilities. **(a):** The TC; **(c):** the VC. In CIC, the red curve (number of high-risk individuals) indicates the number of people who are classified as positive (high risk) by the model at each threshold probability; the blue curve (number of high-risk individuals with event) is the number of true positives at each threshold probability. **(b):** The TC; **(d):** the VC. NDOC patients: newly diagnosed ovarian cancer patients; TC: training cohort. VC: validation cohort.

The established nomogram revealed C-index or AUC of 0.764 (95%CI: 0.744-0.783), and 0.759 (95%CI: 0.731-0.788) in the TC and VC, respectively (Fig. 2a-b). Hosmer-Lemeshow statistics were 9.04 (p = 0.434) and 8.08 (p = 0.537), for the TC and VC respectively. The resulting calibration plots indicated good consistency between the prediction and actual observation (Fig. 2c-d). DCA curves both in the TC and VC addressed that our nomogram had a favorable clinical utility to predict LM development in NDOC patients within the threshold probabilities between 2% and 40% (Fig. 3a, c). CIC analysis visually indicated that nomogram conferred high clinical net benefit (Fig. 3b, d).

### Prognostic factors for OCLM patients

Baseline characteristics for OCLM patients were comparable between the TC and VC (Table s4). The 1-, 3-, 5- year OS rate for OCLM patients was 48.03%, 26.75%, and 15.39%, respectively (Supplementary Figure 3). NDOC patients with and without LM recorded median OS of 11 months (95% CI: 10-13 months) and 55 months (95% CI: 53-57 months), respectively (Supplementary Figure 4a). Univariate Cox regression revealed that age, race, marital status, histology, tumor laterality, T stage, LBB metastases and primary CRS were significant prognostic factors in the TC (*n* = 1,775) (Table s5). The final prognostic nomogram, after backward stepwise selection based on AIC, revealed a significant correlation between increased all-cause mortality with age (HR,1.01; 95% CI: 1.00-1.02; *p* = 0.023), Asian race (vs. White; HR,1.69; 95% CI: 1.15-2.48; *p* = 0.008), Black race (vs. White; HR,1.35; 95% CI: 0.99-1.83; *p* = 0.055), unmarried status (vs. married status; HR, 1.31; 95% CI: 1.07-1.62; *p* = 0.009), non-serous OC (vs. serous OC; HR, 1.70; 95% CI: 1.36-2.11; *p* < 0.001), LBB metastases (vs. no other distant metastases; HR, 1.30; 95% CI: 1.04-1.62; *p* = 0.022), suboptimal CRS (vs. optimal CRS; HR, 1.60; 95%CI: 1.26-2.05; *p* = 0.001), and no CRS (vs. optimal CRS; HR, 4.29; 95% CI: 3.26-5.66; *p* < 0.001). Moreover, primary CRS was the most significant contributor to prognosis, followed by race and histology, whereas LBB metastases had a relatively low impact on patient survival (Fig. 1b). The median OS for the no, suboptimal, and optimal CRS groups were 2 (95% CI: 2-3), 28 (95% CI: 23-36), and 44 (95%CI: 38-53) months, respectively (Table 3, Supplementary Figure 4f). Results from survival analyses targeting other variables in the TC based on Kaplan-Meier (K-M) curves are presented in Supplementary Figure 4b-e. The pooled HR derived from 5 MI datasets was comparable to HR of the complete dataset (Table 3).

### The nomogram model predicts prognosis of OCLM patients

**Figure 4:**
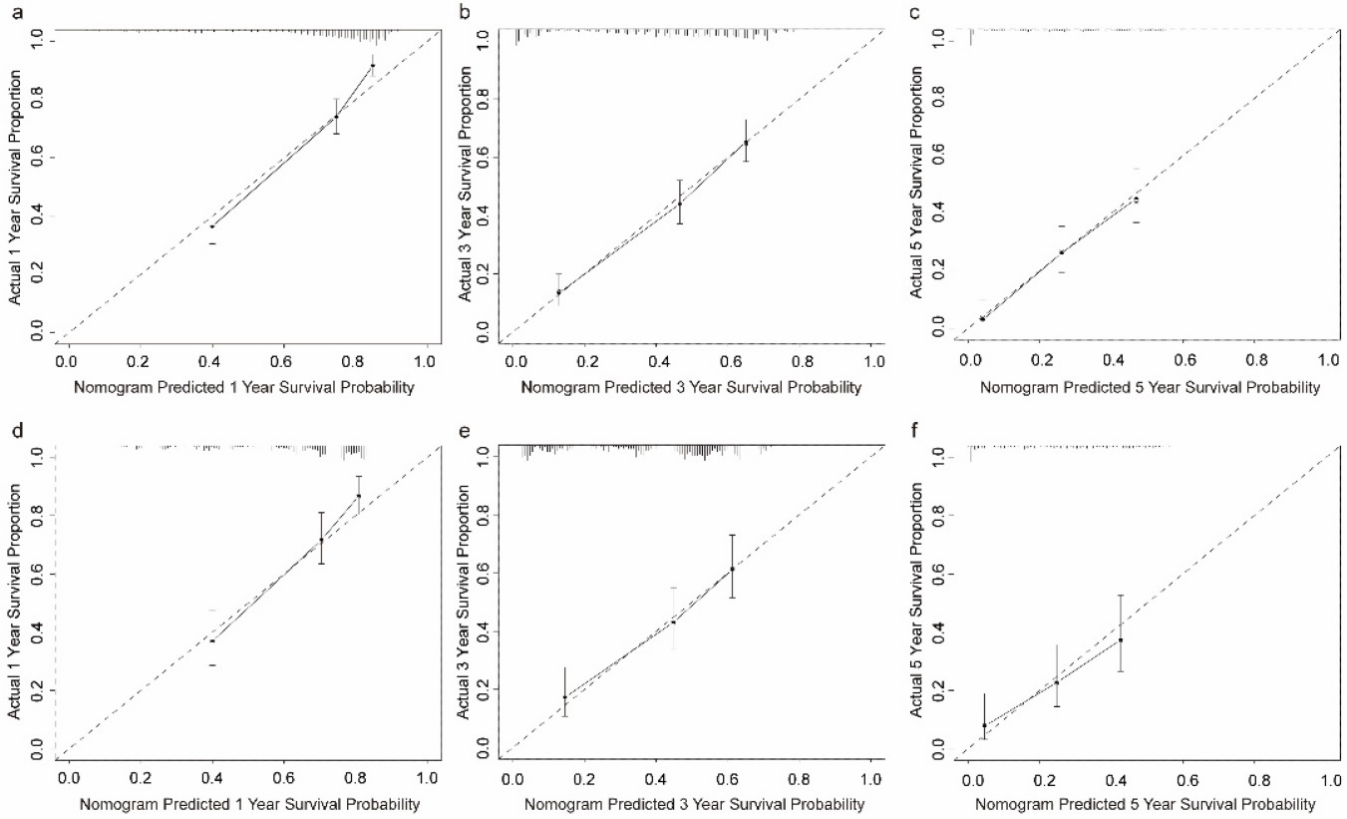
** Calibration plots of the prognostic nomogram for OCLM patients at 1-, 3-, and 5-year survival rates. (a-c):** The training cohort. **(d-f):** The validation cohort. OCLM patients: newly diagnosed ovarian cancer patients with liver metastases.

**Figure 5:**
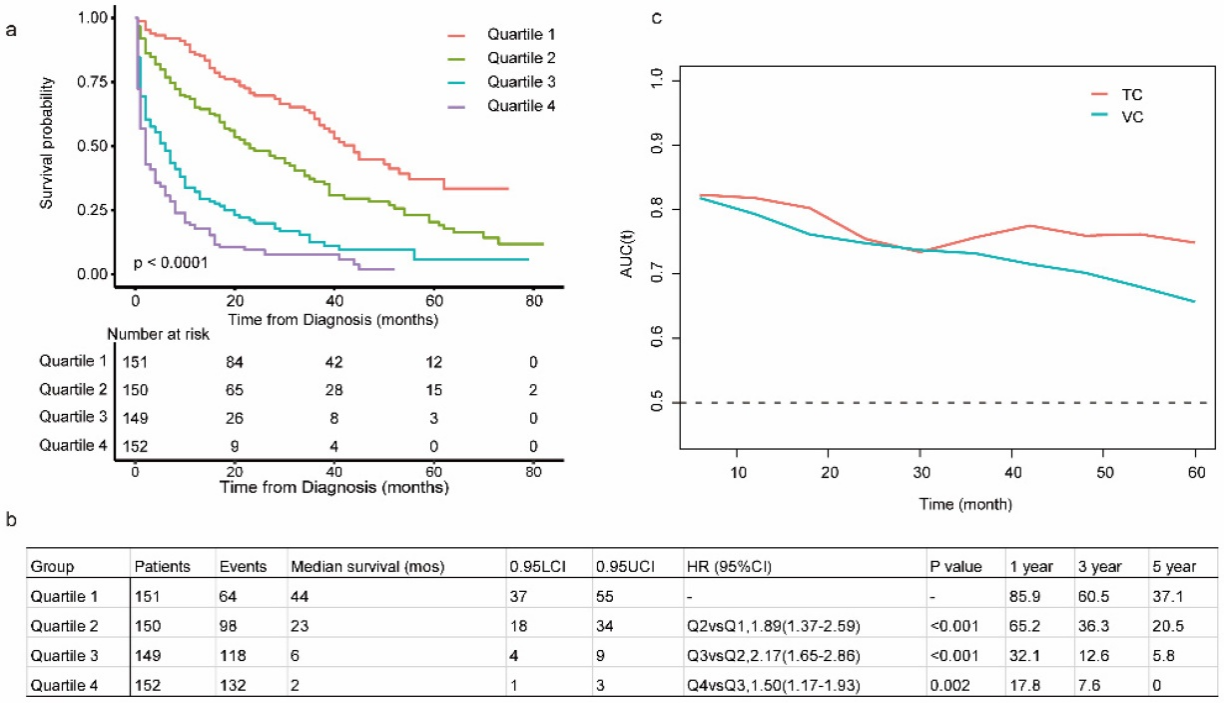
** Evaluation of the prognostic nomogram in the VC of OCLM patients. (a):** Risk scores from cases in the VC were calculated according to the model in Figure 1b and grouped into quartiles. Kaplan-Meier plots are depicted for each group. **(b):** Summary of Quartile 1, 2, 3 and 4. **(c):** Area under the curve was calculated for every month from the first to the 60^th^ month for both TV and VC. LCI and UCI: lower and upper confidence interval. 1-, 3-, 5- year: 1-, 3-, 5- year survival probability. OCLM patients: newly diagnosed ovarian cancer patients with liver metastases. TC: training cohort. VC: validation cohort.

**Figure 6:**
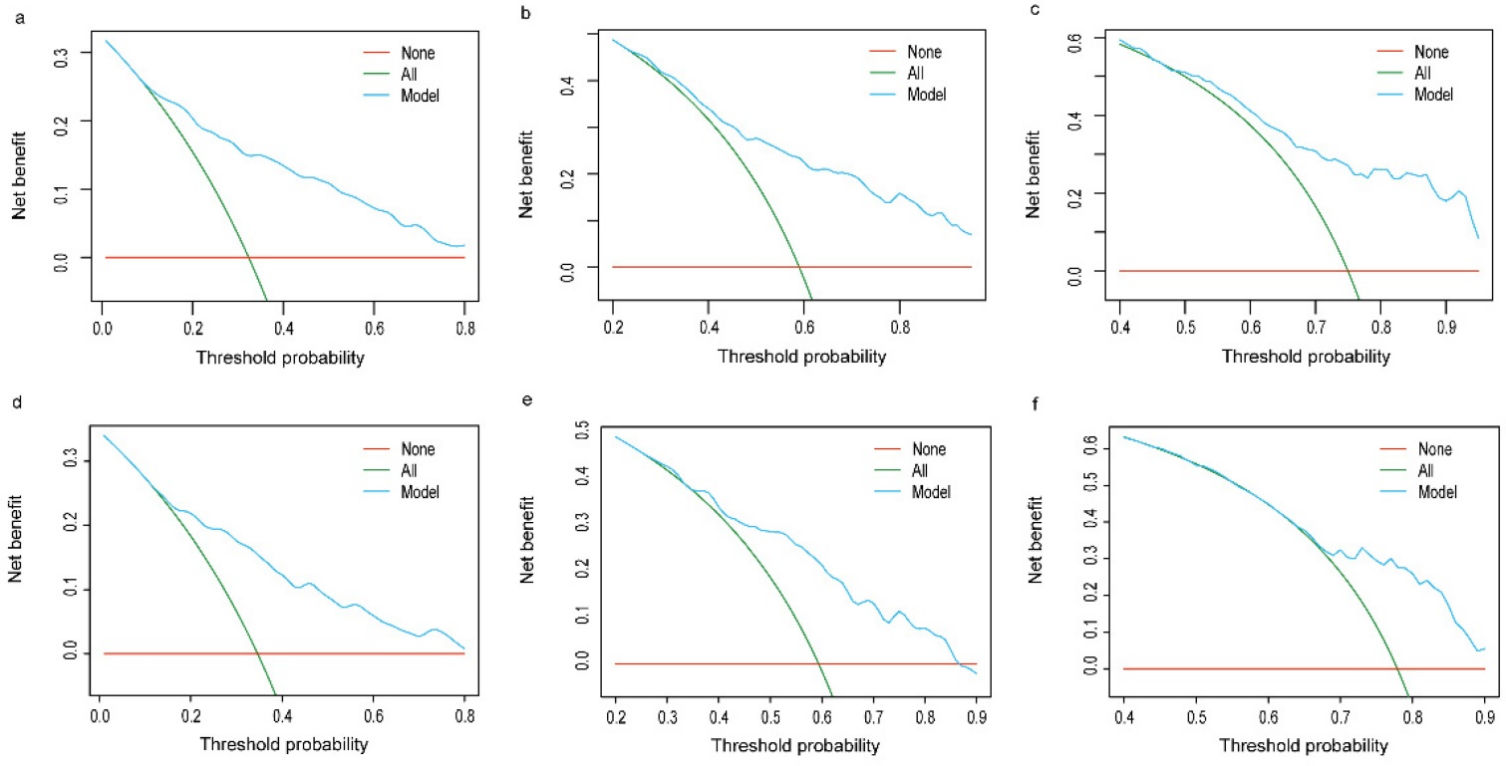
** Decision curve analysis of prognostic nomogram for OCLM patients.** The net benefits (y axis) of prognostic nomogram were calculated for both the training and validation cohorts. **(a):** 12-month OS of TC; **(b):** 36-month OS of TC; **(c):** 60-month OS of TC; **(d):** 12-month OS of VC; **(e):** 36-month OS of VC; **(f):** 60-month OS of VC. Horizontal red lines assume no cases will experience the event; green lines assume all cases will experience the event; blue lines represent the net benefits across a range of threshold probabilities. OCLM patients: newly diagnosed ovarian cancer patients with liver metastases. TC: training cohort. VC: validation cohort.

The C-index in the TC and VC were 0.743 (95% CI: 0.719-0.767) and 0.714 (95% CI: 0.678-0.752), respectively. Calibration curves for the TC and VC at 1-, 3-, and 5-years indicated excellent consistency between prediction and actual observation (Fig. 4). We used the predictive scores to group patients in the VC (*n* = 916) into quartiles. The resulting K-M curves revealed significant prognostic differences between any two adjacent groups (Fig. 5a), with median OS of 44 (95%CI: 37-55), 23 (95%CI: 18-34), 6 (95%CI: 4-9), and 2 (95%CI: 1-3) months for Quartiles 1, 2, 3, and 4, respectively. The 1-, 3-, and 5-year survival probability for Quartiles 1, 2, 3, and 4 were 85.9, 60.5, 37.1%; 65.2, 36.3, 20.5%; 32.1, 12.6, 5.8%; and 17.8, 7.6, 0%, respectively (Fig. 5b). The AUC values of td-ROC were calculated (Fig. 5c), integrated AUC for the TC and VC were 0.773 and 0.733, respectively. DCA curves of the TC and VC at 12-, 36- and 60-month addressed that using the model to inform clinical decisions would lead to superior outcomes over a wide range of threshold probability (Fig. 6).

## Discussion

Missing data is an inevitable and critical question for population-based database like SEER 14, which might result in considerable bias due to improper management 15. Unfortunately, it has been neglected in most SEER studies. The types of missing data included missing completely at random (MCAR), missing at random (MAR), and missing not at random (MNAR) 16. Complete case analysis in the study of Iftikhar et al. is on the assumption of MCAR 7, while the patterns of missing data are MAR in most clinical studies 17. Missing indicator method in the study of Zhao et al. 5 still subject to bias even under MCAR assumption and a small amount of missing data 18. Multiple imputation has been recognized as the standard method to handle missing data with the pattern of MAR 19, which also applies to MCAR and MNAR 17. The amount of missing data for different variables in SEER ranges from 0 to 36.11% in NDOC patients and ranges from 0 to 56.93% in OCLM patients, which is noticeable. However, unbiased results can be obtained after valid MI for MAR data even with up to 80%-90% missing data 20. The pattern of missing data in our study was categorized as MAR by visual inspection, which means the missing information depends on the data we have already collected 15. As a sensitivity analysis, distributions of missing variables were the same in complete cases data and for MI datasets. Moreover, the coefficients of regression analysis from both datasets are comparable. Collectively, selection bias based on complete data in our study is fully considered and limited.

Prior studies with small sample sizes have reported a 9.4-12.9% incidence of LM in OC patients 21,22. Based on population study, Zhao et al. found 1774 OCLM patients in 26197 NDOC patients (6.7%) 5, while Iftikhar et al. reported 2635 OCLM patients in 33895 NDOC patients (7.77%) 7. In the present study, we analyzed 37,500 NDOC patients from the SEER database and found a 7.18% LM incidence.

The liver was the most common distant metastatic organ of OC 4-6, potentially by transcoelomic and hematogenous dissemination route 23, whose underlying mechanisms are less comprehensively and less well understood. Liver metastases could be detected effectively with the development of imaging technology, whereas there are no screening guidelines for LM development in NDOC patients to date. Therefore, timely diagnosis and improved prognosis can be realized by identification and surveillance of high-risk groups. Regrettably, the tool quantifying risk of LM development is not available. Earlier studies focusing on OC with bone and brain metastases suggested that advanced T stage, N stage, tumor grade, and metastases to other distant organs were predictive factors for bone and brain metastases 9,24. Previous population-based studies determined older age, race, unmarried status, bilateral tumor location, non-serous histology, advanced T and N stages, grades, and elevated CA-125 were risks for liver metastases 5,7, our study supported this association. Our risk nomogram had a favorable C-index in both TC and VC, suggesting good discrimination. Moreover, the Hosmer-Lemeshow statistics revealed a good fit, whereas calibration plots indicated consistency between predicted and observed results. DCA curves both in the TC and VC addressed that our nomogram added value to predict LM development in NDOC patients within the threshold probabilities between 2% and 40%. CIC analysis visually indicated that our risk nomogram conferred high clinical net benefit. The nomogram is expected to facilitate risk stratification and to develop an intensive screening program for high-risk groups to aid timely diagnosis and better prognosis.

Despite the 5-year OS plunges from 92% for localized cases to 29% for distant cases 1, aggressive liver resection had been increasingly applied in the recent past, and the favorable surgical prognosis has been confirmed 25-28. Based on a population-level study, we found the median OS of OCLM patients dramatically improved from 2 months in the non-surgery group to 44 months in the optimal CRS group, consistent with previous reports 29,30. Since most advanced OC patients experience a relapse within years after front-line treatment based on surgery and chemotherapy 31, anti-angiogenic therapy 32, poly-ADP ribose polymerase inhibitors (PARPi) 33,34 and immunotherapy 35,36 are ongoing randomized trials as a secondary treatment to improve their outcomes. However, prognosis of OCLM patients varied considerably (IQR of OS: 1.0 to 39.0 months in our study, IQR of OS: 3.0-50.0 months in Zhao et al.'s report). Thus, stratifying OCLM patients with comparable expected prognoses in randomized studies could control selection bias and optimize conclusion. Our study identified age, race, marital status, histological type, LBB metastases and primary CRS were indicators of all-cause mortality in multivariable analysis after stepwise selection, in accordance with previous studies 5,7. Surgical treatment was the most significant prognostic contributor to our nomogram, moreover, secondary CRS is feasible for recurrent OC patients with LM with BRCA mutation for superior progression free survival 37, further affirming the value of CRS in survival improvement for OCLM patients. Although OC patients with brain or bone metastases were found to exhibit a worse prognosis than those with LM 4. LBB metastases had a less prognostic contribution to our nomogram model, possibly due to the low incidence in NDOC patients. The status of lymph node was not an indicator for prognosis in our nomogram, concurred with no prognostic differences between advanced OC patients undergoing CRS with or without hepatoceliac lymph node metastases 38. The resulting C index indicated good discrimination and calibration plots in both TC and VC revealed favorable consistency between prediction and observation. The integrated AUC of td-ROC in TV and VC were favorable. The K-M curves, based on predictive scores from the nomogram, demonstrated that our model could stratify OCLM patients into subgroups of statistically significant prognosis. DCA curves of the TC and VC at 12-, 36- and 60-month addressed that clinical decision based on our nomogram would lead to superior survival over a wide range of threshold probability. Overall, these results indicate that our prognostic nomogram is clinically valuable for individual prediction as well as to stratify OCLM patients in randomized studies.

To our knowledge, the current study was the first largest study to establish nomograms to predict LM development in NDOC patients and to evaluate prognosis of OCLM patients after controlling potential bias from missing data in SEER. However, our work definitely had several limitations. Firstly, we only explored the epidemiology of LM in NDOC patients, for recurrent OC patients were not recorded in SEER. Therefore, the overall LM incidence needs further evaluation. Secondly, information about peritoneal metastases, which is the prominent dissemination route of OC 23, is not available in SEER database. Thirdly, information about chemotherapy is not available for us in SEER. Furthermore, both nomograms have so far been only validated internally and further studies are needed to validate our findings.

## Conclusion

Based on the largest dataset to date in which selection bias was fully considered and limited, our results revealed that the prevalence of LM is about 7.18% in NDOC patients, and OCLM patients exhibited a median OS of 11 months wtih1-, 3-, 5- year OS rate of 48.03%, 26.75%, and 15.39%, respectively. Furthermore, we established a risk nomogram that can effectively predict high-risk groups for LM development in NDOC patients, and aid intensive screening as well as timely diagnosis. We established another prognostic nomogram to facilitate individualized precise prediction and stratification in OCLM patients. However, further studies are required to validate our findings.

## Supplementary Material

Supplementary figures and tables.

## Figures and Tables

**Table 1:** Sociodemographic and clinical characteristics for NDOC patients with and without LM

Variable	No. of ovarian cancer patients (2010-2016)		X^2^	P-value
	With LM (N=2691, 7.18%)	Without LM (N=34809, 92.82%)		
**Age** (median, IQR)^a^	66 (56-76)	62 (52-72)		<0.001
**Race**			68.28	<0.001
White	1799 (7.03%)	23782 (92.97%)		
Hispanic	333 (6.56%)	4742 (93.44%)		
Black	346 (10.56%)	2930 (89.44%)		
Asian	189 (5.88%)	3028 (94.12%)		
unknown	24 (6.84%)	327 (93.16%)		
**Marital status**			38.32	<0.001
Married	1172 (6.37%)	17225 (93.63%)		
Unmarried	1401 (8.06%)	15976 (91.94%)		
Unknown	118 (6.84%)	1608 (93.16%)		
**Insurance**			2.01	0.160
Insured	2535 (7.13%)	32999 (92.87%)		
Uninsured	108 (8.16%)	1216 (91.84%)		
Unknown	48 (7.48%)	594 (92.52%)		
**Ca125**			157.45	<0.001
Normal	70 (2.07%)	3306 (97.93%)		
Elevated	2050 (8.08%)	23314 (91.92%)		
Unknown	571 (6.52%)	8189 (93.48%)		
**Grade**			181.04	<0.001
I-II	124 (1.89%)	6439 (98.11%)		
III	629 (6.41%)	9183 (93.59%)		
IV	406 (5.35%)	7177 (94.65%)		
Unknown	1532 (11.31%)	12010 (88.69%)		
**Histology**			0.62	0.430
Serous	1139 (6.54%)	16273 (93.46%)		
Non-serous	1099 (6.33%)	16250 (93.67%)		
Unknown	453 (16.54%)	2286 (83.46%)		
**Laterality**			134.74	<0.001
Left	445 (4.52%)	9394 (95.48%)		
Right	462 (4.56%)	9662 (95.44%)		
Bilateral	891 (7.69%)	10703 (92.31%)		
Unknown	893 (15.03%)	5050 (84.97%)		
**T stage**			788.60	<0.001
T1	120 (1.18%)	10090 (98.82%)		
T2	203 (4.12%)	4727 (95.88%)		
T3	1721 (9.21%)	16962 (90.79%)		
Unknown	647 (17.60%)	3030 (82.40%)		
**N stage**			467.08	<0.001
N0	1206 (4.62%)	24870 (95.38%)		
N1	867 (11.39%)	6744 (88.61%)		
Unknown	618 (16.21%)	3195 (83.79%)		
**LBB Met**			2223.58	<0.001
None	1794 (5.18%)	32824 (94.82%)		
≥1 site	742 (29.92%)	1738 (70.08%)		
Unknown	155 (38.56%)	247 (61.44%)		
**CRS (pri)**			1662.29	<0.001
Yes	1269 (4.32%)	28082 (95.68%)		
No	1392 (17.64%)	6499 (82.36%)		
Unknown	30 (11.63%)	228 (88.37%)		

**Abbreviations**: NDOC: newly diagnosed ovarian cancer; LM: liver metastases; IQR: interquartile range; LBB Met: lung, bone, or brain metastases; CA125: cancer antigen 125; a: t test; CRS (pri): primary cytoreduction surgery.

**Table 2:** Univariate and multivariate logistic regression identified the risk factors for development of LM in the TC of NDOC patients based on complete data and MI datasets

Subject characteristics	Univariate (complete data)		Multivariate (complete data)		Multivariate (pooled MI data)	
	OR (95%CI)	P-value	OR (95%CI)	P-value	OR (95%CI)	P-value
**Age**	1.02 (1.02, 1.03)	<0.001				
**Marital status**						
Married	1		1		1	
Unmarried	1.28 (1.16, 1.41)	<0.001	1.19 (0.98, 1.45)	0.084	1.19 (1.08, 1.32)	<0.001
**Insurance**						
Insured	1					
Uninsured	1.18 (0.92, 1.51)	0.19				
**Ca125**						
Normal	1					
Elevated	4.08 (3.03, 5.49)	<0.001				
**Histology**						
Serous	1		1		1	
Non-serous	1.02 (0.92, 1.14)	0.692	1.28 (1.02,1.61)	0.053	1.57 (1.41, 1.76)	<0.001
**Laterality**						
Left	1					
Right	1.01 (0.86, 1.19)	0.908				
Bilateral	1.80 (1.56, 2.08)	<0.001				
**Race**						
White	1					
Hispanic	0.93 (0.80, 1.08)	0.335				
Black	1.54 (1.33, 1.79)	<0.001				
Asian	0.77 (0.63, 0.94)	0.01				
**Grade**						
I-II	1		1		1	
III	3.64 (2.85, 4.63)	<0.001	1.61 (1.15, 2.26)	0.004	1.18 (0.99, 1.41)	0.066
IV	2.92 (2.26, 3.76)	<0.001	1.42 (1.01, 2.01)	0.048	1.04 (0.86, 1.27)	0.676
**T stage**						
T1	1		1		1	
T2	3.91 (2.96, 5.18)	<0.001	2.60 (1.48, 4.56)	0.002	2.72 (2.11, 3.51)	<0.001
T3	8.75 (6.93, 11.04)	<0.001	7.50 (4.66, 12.06)	<0.001	5.20 (4.21, 6.43)	<0.001
**N stage**						
N0	1		1		1	
N1	2.65 (2.37, 2.96)	<0.001	1.58 (1.29, 1.93)	<0.001	1.60 (1.43, 1.79)	<0.001
**LBB Met**						
None	1		1		1	
≥1 site	8.28 (7.35, 9.34)	<0.001	6.69 (5.12, 8.73)	<0.001	5.60 (4.93, 6.36)	<0.001

**Abbreviations:** OR: odds ratio; CI: confidence interval; LM: liver metastases; TC: training cohort, NDOC: newly diagnosed ovarian cancer; LBB Met: lung, bone, or brain metastases; CA125: cancer antigen 125; MI: multiple imputation.

**Table 3:** Multivariable cox regression and overall survival for OCLM patients in the TC based on complete data and MI datasets

Subject characteristics	Multivariable (complete data)		Multivariable (pooled MI datasets)		OS, month
	HR (95%CI)	P-value	HR (95%CI)	P-value	Median (95%CI)
**Age**	1.01 (1.00, 1.02)	0.023	1.01 (1.00, 1.02)	0.019	
**Race**					
White	1				13 (11, 15)
Hispanic	0.84 (0.61, 1.15)	0.27	0.91 (0.78, 1.06)	0.229	21 (13, 25)
Black	1.35 (0.99, 1.83)	0.055	1.20 (1.05, 1.38)	0.009	6 (5, 7)
Asian	1.69 (1.15, 2.48)	0.008	1.19 (0.99, 1.44)	0.068	10 (6, 24)
**Marital status**					
Married	1				19 (15, 22)
Unmarried	1.31 (1.07, 1.62)	0.009	1.24 (1.12, 1.37)	<0.001	7 (6, 9)
**Histology**					
Serous	1				30 (27, 37)
Non-serous	1.70 (1.36, 2.11)	<0.001	1.45 (1.31, 1.61)	<0.001	6 (5, 7)
**LBB Met**					
None	1				15 (13, 18)
≥1 site	1.30 (1.04, 1.62)	0.022	1.15 (1.04, 1.28)	0.007	6 (5, 9)
**CRS (pri)**					
optimal	1				44 (38, 53)
suboptimal	1.60 (1.26, 2.05)	<0.001	1.46 (1.24, 1.71)	<0.001	28 (23, 36)
no CRS	4.29 (3.26, 5.66)	<0.001	4.17 (3.57, 4.87)	<0.001	2 (2, 3)

**Abbreviations**: HR: hazard ratio; CI: confidence interval; OCLM patients: newly diagnosed ovarian cancer patients with liver metastases; LBB Met: lung, bone, or brain metastases; CRS (pri): primary cytoreduction surgery. MI: multiple imputation.

## Abbreviations

NDOC: newly diagnosed ovarian cancer
LM: liver metastases
OCLM: NDOC patients with LM
Surveillance, SEER: Epidemiology, and End Results program
MI: multiple imputation

## References

[1] Siegel RL, Miller KD, Jemal A (2019). Cancer statistics, 2019. CA Cancer J Clin.

[2] Cannistra SA (2004). Cancer of the ovary. N Engl J Med.

[3] Prat J (2015). Abridged republication of FIGO's staging classification for cancer of the ovary, fallopian tube, and peritoneum. Cancer.

[4] Deng K, Yang C, Tan Q, Song W, Lu M, Zhao W (2018). Sites of distant metastases and overall survival in ovarian cancer: A study of 1481 patients. Gynecol Oncol.

[5] Zhao H, Xu F, Li J, Ni M, Wu X (2020). A Population-Based Study on Liver Metastases in Women With Newly Diagnosed Ovarian Cancer. Frontiers in oncology.

[6] Gardner AB, Charo LM, Mann AK, Kapp DS, Eskander RN, Chan JK (2020). Ovarian, uterine, and cervical cancer patients with distant metastases at diagnosis: most common locations and outcomes. Clin Exp Metastasis.

[7] Hussain I, Xu J, Deng K, Noor Ul A, Wang C, Huang Y (2020). The Prevalence and associated Factors for Liver Metastases, Development and Prognosis in newly diagnosed Epithelial Ovarian Cancer: A large Population-Based Study from the SEER Database. Journal of Cancer.

[8] White IR, Royston P, Wood AM (2011). Multiple imputation using chained equations: Issues and guidance for practice. Statistics in medicine.

[9] Zhang C, Guo X, Peltzer K, Ma W, Qi L, Zhang Y (2019). The prevalence, associated factors for bone metastases development and prognosis in newly diagnosed ovarian cancer: a large population based real-world study. Journal of Cancer.

[10] Harrell FE, Lee KL, Mark DB (1996). Multivariable prognostic models: issues in developing models, evaluating assumptions and adequacy, and measuring and reducing errors. Statistics in medicine.

[11] Su Y-S, Gelman A, Hill J, Yajima M (2011). Multiple Imputation with Diagnostics (mi) in R: Opening Windows into the Black Box. Journal of Statistical Software.

[12] Li J, Ma S (2011). Time-dependent ROC analysis under diverse censoring patterns. Statistics in medicine.

[13] Howlader N, Noone A-M, Yu M, Cronin KA (2012). Use of imputed population-based cancer registry data as a method of accounting for missing information: application to estrogen receptor status for breast cancer. Am J Epidemiol.

[14] Elliott SP, Johnson DP, Jarosek SL, Konety BR, Adejoro OO, Virnig BA (2012). Bias due to missing SEER data in D'Amico risk stratification of prostate cancer. J Urol.

[15] Jeong CW, Washington SL, Herlemann A, Gomez SL, Carroll PR, Cooperberg MR (2020). The New Surveillance, Epidemiology, and End Results Prostate with Watchful Waiting Database: Opportunities and Limitations. Eur Urol.

[16] Donders ART, van der Heijden GJMG, Stijnen T, Moons KGM (2006). Review: a gentle introduction to imputation of missing values. J Clin Epidemiol.

[17] Pedersen AB, Mikkelsen EM, Cronin-Fenton D, Kristensen NR, Pham TM, Pedersen L (2017). Missing data and multiple imputation in clinical epidemiological research. Clin Epidemiol.

[18] Greenland S, Finkle WD (1995). A critical look at methods for handling missing covariates in epidemiologic regression analyses. Am J Epidemiol.

[19] Hoskin TL, Boughey JC, Day CN, Habermann EB (2019). Lessons Learned Regarding Missing Clinical Stage in the National Cancer Database. Annals of surgical oncology.

[20] Madley-Dowd P, Hughes R, Tilling K, Heron J (2019). The proportion of missing data should not be used to guide decisions on multiple imputation. J Clin Epidemiol.

[21] Cormio G, Rossi C, Cazzolla A, Resta L, Loverro G, Greco P (2003). Distant metastases in ovarian carcinoma. Int J Gynecol Cancer.

[22] Dauplat J, Hacker NF, Nieberg RK, Berek JS, Rose TP, Sagae S (1987). Distant metastases in epithelial ovarian carcinoma. Cancer.

[23] Barbolina MV (2018). Molecular Mechanisms Regulating Organ-Specific Metastases in Epithelial Ovarian Carcinoma. Cancers (Basel).

[24] Xi S, Li Z, Guo Q, Lin W, Liang X, Ma L (2020). Prognostic Factors among Brain Metastases in Newly Diagnosed Ovary Cancer: A Large Real-world Study. Journal of Cancer.

[25] Bacalbasa N, Dima S, Brasoveanu V, David L, Balescu I, Purnichescu-Purtan R (2015). Liver resection for ovarian cancer liver metastases as part of cytoreductive surgery is safe and may bring survival benefit. World journal of surgical oncology.

[26] Pekmezci S, Saribeyoglu K, Aytac E, Arvas M, Demirkiran F, Ozguroglu M (2010). Surgery for isolated liver metastasis of ovarian cancer. Asian J Surg.

[27] Bacalbaşa N, Balescu I, Dima S, Popescu I (2015). Long-term Survivors After Liver Resection for Ovarian Cancer Liver Metastases. Anticancer research.

[28] Wang M, Zhou J, Zhang L, Zhao Y, Zhang N, Wang L (2019). Surgical treatment of ovarian cancer liver metastasis. Hepatobiliary Surg Nutr.

[29] Liu PC, Benjamin I, Morgan MA, King SA, Mikuta JJ, Rubin SC (1997). Effect of surgical debulking on survival in stage IV ovarian cancer. Gynecol Oncol.

[30] Curtin JP, Malik R, Venkatraman ES, Barakat RR, Hoskins WJ (1997). Stage IV ovarian cancer: impact of surgical debulking. Gynecol Oncol.

[31] Reverdy T, Sajous C, Péron J, Glehen O, Bakrin N, Gertych W, Lopez J, You B, Freyer G (2020). Front-Line Maintenance Therapy in Advanced Ovarian Cancer-Current Advances and Perspectives. Cancers (Basel).

[32] Tewari KS, Burger RA, Enserro D, Norquist BM, Swisher EM, Brady MF (2019). Final Overall Survival of a Randomized Trial of Bevacizumab for Primary Treatment of Ovarian Cancer. Journal of clinical oncology: official journal of the American Society of Clinical Oncology.

[33] Moore KN, Secord AA, Geller MA, Miller DS, Cloven N, Fleming GF (2019). Niraparib monotherapy for late-line treatment of ovarian cancer (QUADRA): a multicentre, open-label, single-arm, phase 2 trial. The Lancet Oncology.

[34] González-Martín A, Pothuri B, Vergote I, DePont Christensen R, Graybill W, Mirza MR (2019). Niraparib in Patients with Newly Diagnosed Advanced Ovarian Cancer. N Engl J Med.

[35] Harter P, Bidziński M, Colombo N, Floquet A, Rubio Pérez MJ, Kim J-W (2019). DUO-O: A randomized phase III trial of durvalumab (durva) in combination with chemotherapy and bevacizumab (bev), followed by maintenance durva, bev and olaparib (olap), in newly diagnosed advanced ovarian cancer patients. Journal of Clinical Oncology.

[36] Vergote I, Sehouli J, Salutari V, Zola P, Madry R, Wenham RM (2019). ENGOT-OV43/KEYLYNK-001: A phase III, randomized, double-blind, active- and placebo-controlled study of pembrolizumab plus chemotherapy with olaparib maintenance for first-line treatment of BRCA-nonmutated advanced epithelial ovarian cancer. Journal of Clinical Oncology.

[37] Gallotta V, Conte C, D'Indinosante M, Capoluongo E, Minucci A, De Rose AM (2019). Prognostic factors value of germline and somatic brca in patients undergoing surgery for recurrent ovarian cancer with liver metastases. European journal of surgical oncology: the journal of the European Society of Surgical Oncology and the British Association of Surgical Oncology.

[38] Gallotta V, Ferrandina G, Vizzielli G, Conte C, Lucidi A, Costantini B (2017). Hepatoceliac Lymph Node Involvement in Advanced Ovarian Cancer Patients: Prognostic Role and Clinical Considerations. Annals of surgical oncology.

