# Supplementary material for: Nomogram Models for Predicting Risk and Prognosis of Newly Diagnosed Ovarian Cancer Patients with Liver Metastases - A Large Population-Based Real-World Study
Source: J Cancer. 2021 Oct 25;12(24):7255–65. doi: 10.7150/jca.64255 (PMC8734403; doi:10.7150/jca.64255)
Supplement: Supplementary file 1 — Supplementary figures and tables. [file jcav12p7255s1.pdf]

Supporting Materials

Table S1. Sociodemographic and clinical characteristics of NDOC patients from complete case data and multiple imputation datasets.

Variable	No. of ovarian cancer patients (2010-2016)						P-value
	Complete data	MI 1	MI 2	MI 3	MI 4	MI 5	
**Age**(median, IQR)	62.0 (52.0-73.0)	62.0 (52.0-73.0)	62.0 (52.0-73.0)	62.0 (52.0-73.0)	62.0 (52.0-73.0)	62.0 (52.0-73.0)	1
**Race**							1
White	25581 (68.86%)	25792 (68.78%)	25796 (68.79%)	25811 (68.83%)	25827 (68.87%)	25815 (68.84%)	
Hispanic	5075 (13.66%)	5138 (13.70%)	5123 (13.66%)	5130 (13.68%)	5115 (13.64%)	5135 (13.69%)	
Black	3276 (8.82%)	3313 (8.83%)	3311 (8.83%)	3308 (8.82%)	3313 (8.83%)	3300 (8.80%)	
Asian	3217 (8.66%)	3257 (8.69%)	3270 (8.72%)	3251 (8.67%)	3245 (8.65%)	3250 (8.67%)	
**Marital status**							0.95
Married	18397 (51.43%)	19192 (51.18%)	19162 (51.10%)	19191 (51.18%)	19151 (51.07%)	19198 (51.19%)	
Unmarried	17377 (48.57%)	18308 (48.82%)	18338 (48.90%)	18309 (48.82%)	18349 (48.93%)	18302 (48.81%)	
**Insurance**							0.91
Insured	35534 (96.41%)	36110 (96.29%)	36094 (96.25%)	36113 (96.30%)	36128 (96.34%)	36129 (96.34%)	
Uninsured	1324 (3.59%)	1390 (3.71%)	1406 (3.75%)	1387 (3.70%)	1372 (3.66%)	1371 (3.66%)	
**Ca125**							0.429
Normal	25364 (88.25%)	32993 (87.98%)	32929 (87.81%)	33030 (88.08%)	33000 (88.00%)	33094 (88.25%)	
Elevated	3376 (11.75%)	4507 (12.02%)	4571 (12.19%)	4470 (11.92%)	4500 (12.00%)	4406 (11.75%)	
**Grade**							0.698
Ⅰ - Ⅱ	6563 (27.39%)	10339 (27.57%)	10440 (27.84%)	10346 (27.59%)	10395 (27.72%)	10204 (27.21%)	
Ⅲ	9812 (40.96%)	15296 (40.79%)	15285 (40.76%)	15353 (40.94%)	15176 (40.47%)	15356 (40.95%)	
Ⅳ	7583 (31.65%)	11865 (31.64%)	11775 (31.40%)	11801 (31.47%)	11929 (31.81%)	11940 (31.84%)	
**Histology**							0.771
Serous	17412 (50.09%)	18937 (50.50%)	18979 (50.61%)	18962 (50.57%)	18954 (50.54%)	18934 (50.49%)	
Non-serous	17349 (49.91%)	18563 (49.50%)	18521 (49.39%)	18538 (49.43%)	18546 (49.46%)	18566 (49.51%)	
**Laterality**							0.115
Left	9839 (31.18%)	11392 (30.38%)	11741 (31.31%)	11693 (31.18%)	11632 (31.02%)	11572 (30.86%)	
Right	10124 (32.08%)	12180 (32.48%)	12113 (32.30%)	12142 (32.38%)	12128 (32.34%)	11970 (31.92%)	
Bilateral	11594 (36.74%)	13928 (37.14%)	13646 (36.39%)	13665 (36.44%)	13740 (36.64%)	13958 (37.22%)	
**T stage**							0.98
T1	10210 (30.19%)	11280 (30.08%)	11324 (30.20%)	11325 (30.20%)	11338 (30.23%)	11395 (30.39%)	
T2	4930 (14.58%)	5506 (14.68%)	5512 (14.70%)	5539 (14.77%)	5565 (14.84%)	5568 (14.85%)	
T3	18683 (55.24%)	20714 (55.24%)	20664 (55.10%)	20636 (55.03%)	20597 (54.93%)	20537 (54.77%)	
**N stage**							0.314
N0	26076 (77.41%)	28770 (76.72%)	28871 (76.99%)	28832 (76.89%)	28831 (76.88%)	28790 (76.77%)	
N1	7611 (22.59%)	8730 (23.28%)	8629 (23.01%)	8668 (23.11%)	8669 (23.12%)	8710 (23.23%)	
**Met to LBB**							1
None	34618 (93.32%)	35003 (93.34%)	34990 (93.31%)	35003 (93.34%)	35004 (93.34%)	35012 (93.37%)	

⩾ 1 site	2480 (6.68%)	2497 (6.66%)	2510 (6.69%)	2497 (6.66%)	2496 (6.66%)	2488 (6.63%)	
**Liver metastasis**							1
No	34809 (92.82%)	34809 (92.82%)	34809 (92.82%)	34809 (92.82%)	34809 (92.82%)	34809 (92.82%)	
Yes	2691 (7.18%)	2691 (7.18%)	2691 (7.18%)	2691 (7.18%)	2691 (7.18%)	2691 (7.18%)	

Abbreviations: NDOC: newly diagnosed ovarian cancer; MI: multiple imputation; IQR: interquartile range; Met to LBB: metastases to lung, bone, or brain; CA125: cancer antigen 125; MI: multiple imputation.

Table S2. Sociodemographic and clinical characteristics of OCLM patients from complete case data and multiple imputation datasets.

Variable	No. of ovarian cancer patients (2010-2016, n=2691)						P-value
	Complete data	MI 1	MI 2	MI 3	MI 4	MI 5	
**Age**(median, IQR)	66.00 (56.50-76.00)	66.00 (56.50-76.00)	66.00 (56.50-76.00)	66.00 (56.50-76.00)	66.00 (56.50-76.00)	66.00 (56.50-76.00)	1
**Race**							1
White	1799 (67.45%)	1810 (67.26%)	1818 (67.56%)	1814 (67.41%)	1815 (67.45%)	1812 (67.34%)	
Hispanic	333 (12.49%)	340 (12.63%)	335 (12.45%)	337 (12.52%)	336 (12.49%)	339 (12.60%)	
Black	346 (12.97%)	350 (13.01%)	347 (12.89%)	348 (12.93%)	349 (12.97%)	348 (12.93%)	
Asian	189 (7.09%)	191 (7.10%)	191 (7.10%)	192 (7.13%)	191 (7.10%)	192 (7.13%)	
**Marital status**							0.98
Married	1172 (45.55%)	1229 (45.67%)	1220 (45.34%)	1216 (45.19%)	1201 (44.63%)	1224 (45.48%)	
Unmarried	1401 (54.45%)	1462 (54.33%)	1471 (54.66%)	1475 (54.81%)	1490 (55.37%)	1467 (54.52%)	
**Insurance**							0.91
Insured	35534 (96.41%)	36110 (96.29%)	36094 (96.25%)	36113 (96.30%)	36128 (96.34%)	36129 (96.34%)	
Uninsured	1324 (3.59%)	1390 (3.71%)	1406 (3.75%)	1387 (3.70%)	1372 (3.66%)	1371 (3.66%)	
**Ca125**							0.766
Normal	2050 (96.70%)	2587 (96.14%)	2582 (95.95%)	2592 (96.32%)	2587 (96.14%)	2597 (96.51%)	
Elevated	70 (3.30%)	104 (3.86%)	109 (4.05%)	99 (3.68%)	104 (3.86%)	94 (3.49%)	
**Grade**							0.98
Ⅰ-Ⅱ	124, (10.70%)	312, (11.60%)	301, (11.20%)	283, (10.50%)	281, (10.43%)	289, (10.75%)	
Ⅲ	629, (54.27%)	1442, (53.57%)	1454, (54.03%)	1462, (54.34%)	1464, (54.43%)	1460, (54.24%)	
Ⅳ	406, (35.03%)	937, (34.83%)	936, (34.77%)	946, (35.16%)	946, (35.14%)	942, (35.01%)	
**Histology**							0.202
Serous	1139 (50.89%)	1266.9228 (47.08%)	1313.7462 (48.82%)	1321.8192 (49.12%)	1323.7029 (49.19%)	1314.8226 (48.86%)	
Non-serous	1099 (49.11%)	1424.0772 (52.92%)	1377.2538 (51.18%)	1369.1808 (50.88%)	1367.2971 (50.81%)	1376.1774 (51.14%)	
**Laterality**							0.551
Left	445 (24.75%)	741 (27.54%)	709 (26.35%)	720 (26.76%)	713 (26.50%)	722 (26.83%)	
Right	462 (25.70%)	717 (26.64%)	737 (27.39%)	707 (26.27%)	701 (26.05%)	719 (26.72%)	
Bilateral	891 (49.56%)	1233 (45.82%)	1245 (46.27%)	1264 (46.97%)	1277 (47.45%)	1250 (46.45%)	
**T stage**							0.753
T1	120 (5.87%)	164 (6.09%)	163 (6.06%)	169 (6.28%)	162 (6.02%)	179 (6.65%)	
T2	203 (9.93%)	281 (10.44%)	289 (10.74%)	303 (11.26%)	314 (11.67%)	281 (10.44%)	
T3	1721 (84.20%)	2246 (83.46%)	2239 (83.20%)	2219 (82.46%)	2215 (82.31%)	2231 (82.91%)	

**N stage**							0.494
N0	1206 (58.18%)	1522 (56.56%)	1538 (57.15%)	1583 (58.83%)	1579 (58.68%)	1547 (57.49%)	
N1	867 (41.82%)	1169 (43.44%)	1153 (42.85%)	1108 (41.17%)	1112 (41.32%)	1144 (42.51%)	
**Met to LBB**							1
None	1794 (70.74%)	1902 (70.68%)	1899 (70.57%)	1904 (70.75%)	1911 (71.01%)	1904 (70.75%)	
⩾ 1 site	742 (29.26%)	789 (29.32%)	792 (29.43%)	787 (29.25%)	780 (28.99%)	787 (29.25%)	
**Insurance**							0.919
Insured	2535 (95.91%)	2566 (95.35%)	2579 (95.84%)	2577 (95.76%)	2578 (95.80%)	2581 (95.91%)	
Uninsured	108 (4.09%)	125 (4.65%)	112 (4.16%)	114 (4.24%)	113 (4.20%)	110 (4.09%)	
**CRS**							1
Optimal	598 (24.41%)	655 (24.34%)	648 (24.08%)	658 (24.45%)	661 (24.56%)	655 (24.34%)	
suboptimal	460 (18.78%)	519 (19.29%)	524 (19.47%)	519 (19.29%)	526 (19.55%)	523 (19.44%)	
no CRS	1392 (56.82%)	1517 (56.37%)	1519 (56.45%)	1514 (56.26%)	1504 (55.89%)	1513 (56.22%)	

Abbreviations: OCLM patients: newly diagnosed ovarian cancer patients with liver metastases; MI: multiple imputation; IQR: interquartile range; Met to LBB: metastases to lung, bone, or brain; CA125: cancer antigen 125; CRS: primary cytoreduction surgery; MI: multiple imputation.

Table s3. Sociodemographic and clinical characteristics of NDOC patients in the TC and VC

	No. of OC patients (2010-2016)		
Variables	Training cohort (N=24718, 66%)	Validation cohort (N=12782, 34%)	P-value
**Age** (median, IQR) [a]	62.00 (52-73)	62 (52-73)	0.808
**Race**			0.165
White	16878 (68.94%)	8703 (68.71%)	
Hispanic	3323 (13.57%)	1752 (13.83%)	
Black	2201 (8.99%)	1075 (8.49%)	
Asian	2080 (8.50%)	1137 (8.98%)	
**Marital status**			0.435
Married	12155 (51.57%)	6242 (51.14%)	
Unmarried	11413 (48.43%)	5964 (48.86%)	
**Insurance**			0.802
Insured	23414 (96.43%)	12120 (96.37%)	
Uninsured	868 (3.57%)	456 (3.63%)	
**Ca125**			0.378
Normal	2202 (11.63%)	1174 (11.98%)	
Elevated	16738 (88.37%)	8626 (88.02%)	
**Grade**			0.613
Ⅰ-Ⅱ	4319 (27.26%)	2244 (27.66%)	
Ⅲ	6524 (41.18%)	3288 (40.52%)	
Ⅳ	5001 (31.56%)	2582 (31.82%)	
**Histology**			0.996
Serous	11479 (50.09%)	5933 (50.09%)	
Non-serous	11437 (49.91%)	5912 (49.91%)	
**Laterality**			0.387

Left	6483 (31.13%)	3356 (31.28%)	
Right	6734 (32.33%)	3390 (31.60%)	
Bilateral	7611 (36.54%)	3983 (37.12%)	
**T stage**			0.787
T1	6711 (30.09%)	3499 (30.38%)	
T2	3268 (14.65%)	1662 (14.43%)	
T3	12327 (55.26%)	6356 (55.19%)	
**N stage**			0.037
N0	17110 (77.07%)	8966 (78.07%)	
N1	5092 (22.93%)	2519 (21.93%)	
**LBB Met**			0.784
None	22813 (93.29%)	11805 (93.36%)	
⩾ 1 site	1641 (6.71%)	839 (6.64%)	
**LM**			0.992
No	22944 (92.82%)	11865 (92.83%)	
Yes	1774 (7.18%)	917 (7.17%)	

Abbreviations: IQR: interquartile range; NDOC: newly diagnosed ovarian cancer; LM: liver metastases; LBB Met: lung, bone, or brain metastases; CA125: cancer antigen 125; TC: training cohort; VC: validation cohort; a: t test. All factors with unknown data were removed.

Table s4. Sociodemographic and clinical characteristics of OCLM patients in the TC and VC

	No. of OCLM patients (2010-2016)			
Variable	Training (N=1775, 66%)	Validation (N=916, 34%)	X^2	P-value
**Age** (median, IQR) [a]				0.710
	66（56-76）	66（57-76）		
**Race**			6.10	0.192
White	1182 (65.70%)	617 (34.30%)		
Hispanic	221 (66.37%)	112 (33.63%)		
Black	243 (70.23%)	103 (29.77%)		
Asian	113 (59.79%)	76 (40.21%)		
Unknown	16 (66.67%)	8 (33.33%)		
**Marital status**			1.57	0.210
Married	788 (67.24%)	384 (32.76%)		
Unmarried	909 (64.88%)	492 (35.12%)		
**Insurance**			1.00	0.318
Insured	1666 (65.72%)	869 (34.28%)		
Uninsured	76 (70.37%)	32 (29.63%)		
**Ca125**			0.01	0.920
Normal	1359 (66.29%)	691 (33.71%)		
Elevated	46 (65.71%)	24 (34.29%)		

**Grade**			0.15	0.930
Ⅰ-Ⅱ	81 (65.32%)	43 (34.68%)		
Ⅲ	422 (67.09%)	207 (32.91%)		
Ⅳ	271 (66.75%)	135 (33.25%)		
**Histology**			0.02	0.884
Serous	763 (66.99%)	376 (33.01%)		
Non-serous	733 (66.70%)	366 (33.30%)		
**Laterality**			1.17	0.558
Left	303 (68.09%)	142 (31.91%)		
Right	299 (64.72%)	163 (35.28%)		
Bilateral	589 (66.11%)	302 (33.89%)		
**T**			4.73	0.094
T1	69 (57.50%)	51 (42.50%)		
T2	140 (68.97%)	63 (31.03%)		
T3	1141 (66.30%)	580 (33.70%)		
**N**			1.26	0.262
N0	813 (67.41%)	393 (32.59%)		
N1	564 (65.05%)	303 (34.95%)		
**LBB Met**			0.56	0.453
None	1198 (66.78%)	596 (33.22%)		
⩾ 1 site	484 (65.23%)	258 (34.77%)		
**CRS (pri)**			0.47	0.789
Optimal	387 (64.72%)	211 (35.28%)		
suboptimal	307 (66.74%)	153 (33.26%)		
no CRS	912 (65.52%)	480 (34.48%)		

Abbreviations: OCLM patients: newly diagnosed ovarian cancer patients with liver metastases; TC: training cohort; VC: validation cohort; IQR: interquartile range; LBB Met: lung, bone, or brain metastases; CRS (pri): primary cytoreduction surgery; CA125: cancer antigen 125; a: t test. All variables with unknown data were removed.

Table s5. Univariable and multivariable cox regression identified prognostic factors for OCLM patients in the TC

Subject	Univariable		Multivariable	
characteristics	HR (95%CI)	P-value	HR (95%CI)	P-value
**Age**	1.03 (1.02, 1.03)	<0.001	1.01(1.01,1.02)	0.023
**Race**				
White	1		1	
Hispanic	0.81 (0.67, 0.97)	0.021	0.84(0.61,1.15)	0.270

Black	1.49 (1.27, 1.74)	<0.001	1.35(0.99,1.83)	0.055
Asian	1.00 (0.78, 1.27)	0.985	1.69(1.15,2.48)	0.008
**Marital status**				
Married	1		1	
Unmarried	1.48 (1.32, 1.66)	<0.001	1.31(1.07,1.62)	0.009
**Insurance**				
Insured	1			
Uninsured	0.98 (0.74, 1.29)	0.871		
**Ca125**				
Normal	1			
Elevated	1.04 (0.73, 1.49)	0.836		
**Grade**				
Ⅰ-Ⅱ	1			
Ⅲ	1.00 (0.73, 1.36)	0.999		
Ⅳ	0.77 (0.56, 1.08)	0.129		
**Histology**				
Serous	1		1	
Non-serous	2.57 (2.26, 2.93)	<0.001	1.70(1.36,2.11)	<0.001
**Laterality**				
Left	1			
Right	0.91 (0.75, 1.11)	0.347		
Bilateral	0.63 (0.53, 0.75)	<0.001		
**T stage**				
T1	1			
T2	0.66 (0.47, 0.92)	0.014		
T3	0.43 (0.33, 0.57)	<0.001		
**N stage**				
N0	1			
N1	0.89 (0.78, 1.02)	0.097		
**LBB Met**				
None	1		1	
⩾ 1 site	1.44 (1.28, 1.63)	<0.001	1.30(1.04,1.62)	0.022
**CRS (pri)**				
optimal	1		1	
suboptimal	1.60 (1.29, 1.99)	<0.001	1.60(1.26,2.05)	<0.001
no CRS	5.93 (4.97, 7.08)	<0.001	4.29(3.26,5.66)	<0.001

Abbreviations: OCLM patients: newly diagnosed ovarian cancer patients with liver metastases; TC: training cohort; HR: hazard ratio; CI: confidence interval; LBB Met: lung, bone, or brain metastases; CRS (pri): primary cytoreduction surgery; CA125: cancer antigen 125. All factors with unknown data were removed in cox regression model.

Figures

Figure s1

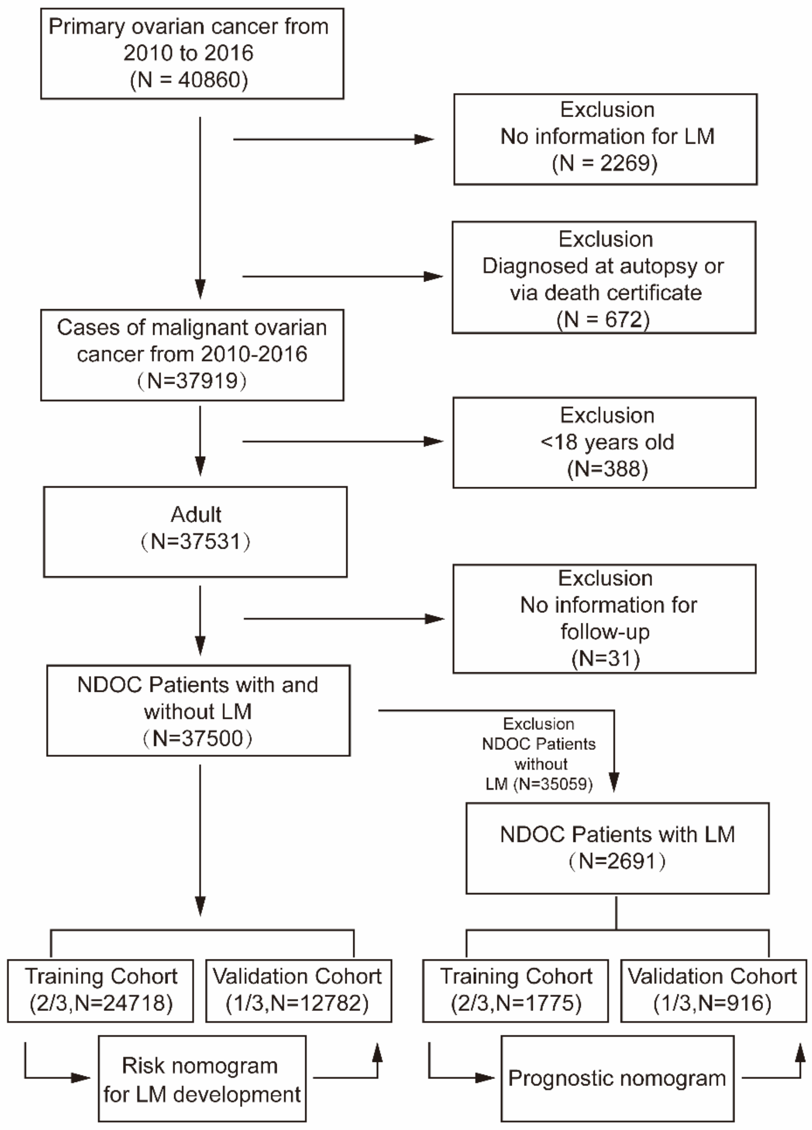

Figure s2

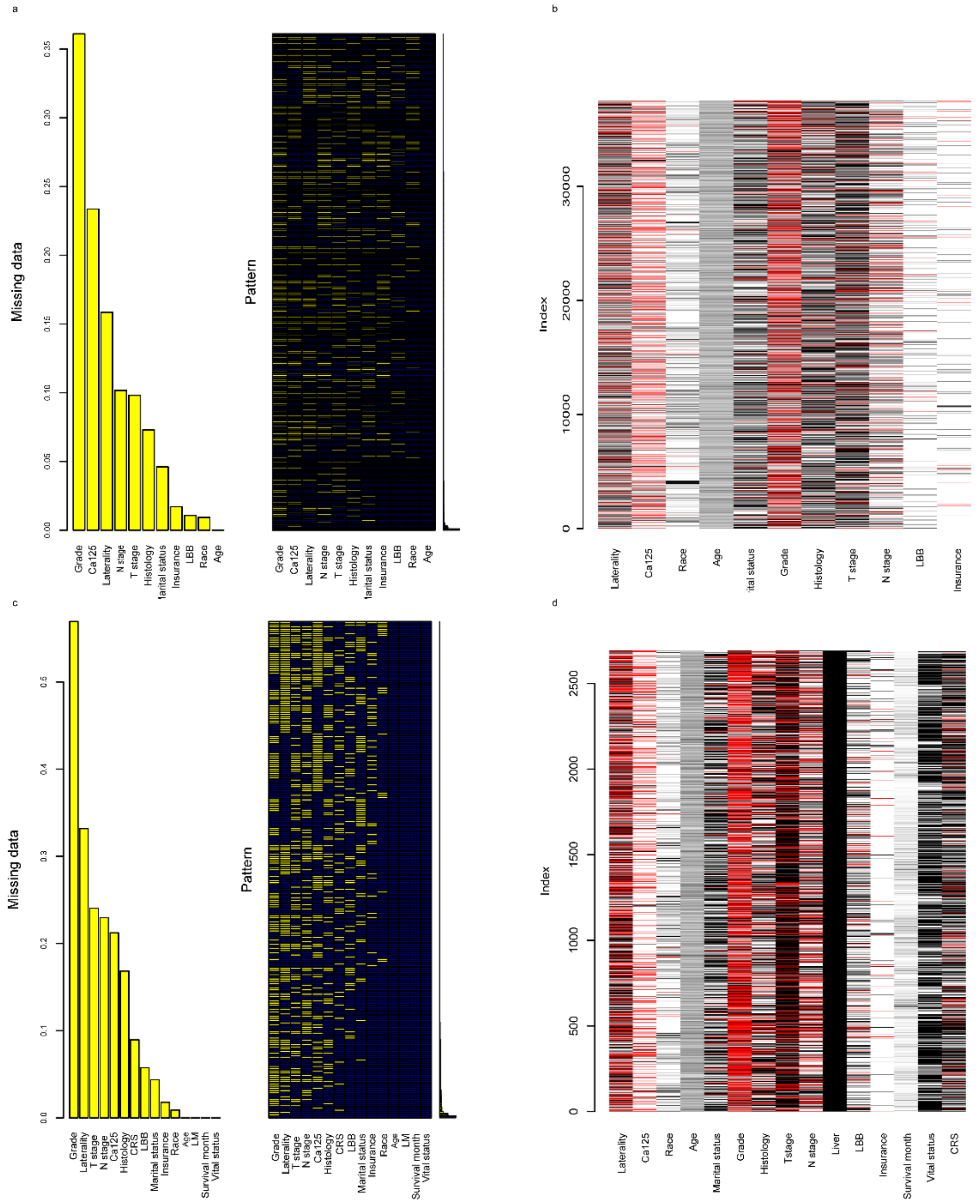
a
Missing data
Pattern
Grade
Ca125
Laterality
N stage
T stage
Histology
Insurance
LBB
Race
Age
b
Index
Race
Age
Grade
Histology
T stage
N stage
c
d
CRS
LM
Survival month
Vital status
Marital status
Tstage
Liver

Figure s3

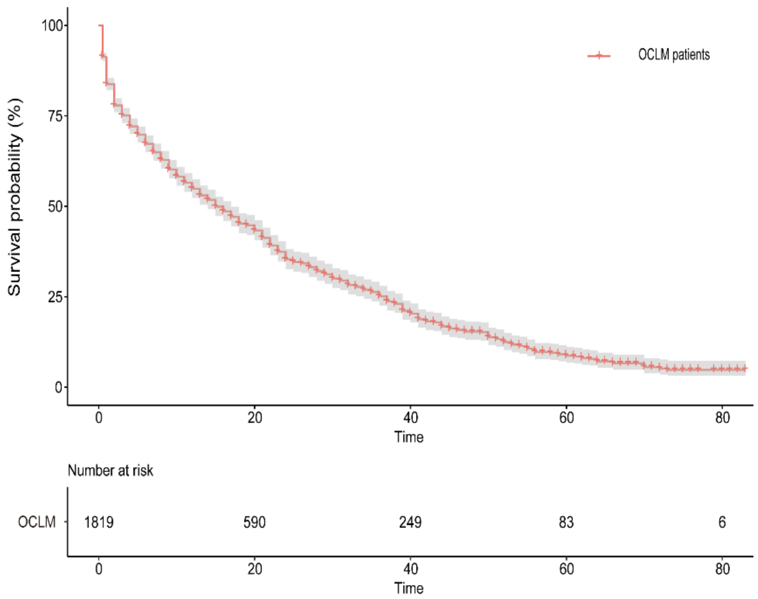

Figure s4

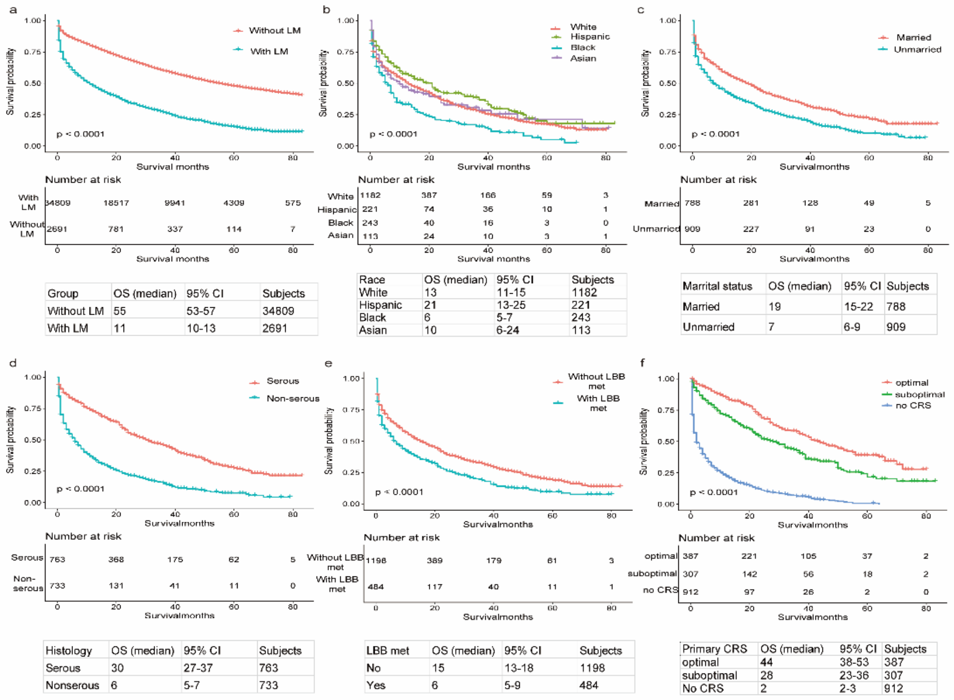

Figure legends

Figure s1. The flow chart of subjects selection and grouping.

NDOC: newly diagnosed ovarian cancer. LM: liver metastases

Figure s2. Visual plotting of missing pattern.

(a): Aggregation plot for missing data of NDOC patients; (b): Matrix plot for missing data of NDOC patients; (c): Aggregation plot for missing data of OCLM patients; (d): Matrix plot for missing data of OCLM patients. NDOC: newly diagnosed ovarian cancer; LM: liver metastases; OCLM patients: NDOC patients with LM. LBB: lung, bone, or brain metastases; CA125: cancer antigen 125.

Figure S3. Overall survival of OCLM patients by Kaplan-Meier curves.

OCLM patients: newly diagnosed ovarian cancer patients with liver metastases.

Figure S4. Survival differences of NDOC patients with and without LM and survival differences among subgroups of variables in OCLM patients.

(a): The Kaplan-Meier curve illustrated survival differences of NDOC patients with and without LM, N=37500; (b-f): The Kaplan-Meier curves demonstrated survival differences of variables which were selected to establish the prognostic nomogram in the TC of OCLM patients, N=1775. (b): Race; (c): Marital status; (d): Histology; (e): LBB metastases; (f): primary CRS. LBB metastases: lung, bone, or brain metastases, CRS: cytoreduction surgery. LM: liver metastases. TC: training cohort. NDOC: newly diagnosed ovarian cancer. LM: liver metastases. OCLM patients: NDOC patients with LM. All factors with unknown data were removed in survival analysis.
